# Dismantling the bacterial virulence program

**DOI:** 10.1111/1751-7915.13388

**Published:** 2019-03-12

**Authors:** Morgan A. Alford, Daniel Pletzer, Robert E.W. Hancock

**Affiliations:** ^1^ Centre for Microbial Diseases and Immunity Research Department of Microbiology and Immunology University of British Columbia Vancouver BC Canada

## Abstract

In the face of rising antimicrobial resistance, there is an urgent need for the development of efficient and effective anti‐infective compounds. Adaptive resistance, a reversible bacterial phenotype characterized by the ability to surmount antibiotic challenge without mutation, is triggered to cope *in situ* with several stressors and is very common clinically. Thus, it is important to target stress‐response effectors that contribute to in vivo adaptations and associated lifestyles such as biofilm formation. Interfering with these proteins should provide a means of dismantling bacterial virulence for treating infectious diseases, in combination with conventional antibiotics.

Antibiotic resistance is one of the biggest threats to the modern medical system since it compromises our ability to perform major, and even minor, procedures without the chance of antagonistic infections. The virulence of opportunistic pathogens, in particular, is mitigated by their growth circumstances, whereby they rapidly adapt to the harsh environmental conditions of the human host. Associated lifestyle changes are dependent on these growth circumstances and trigger complex adaptation mechanisms including adaptive resistance to stressors that are particularly challenging to address. The host milieu is characterized by the presence of various stressors, including temperature changes, oxygen and nutrient (iron, weak nitrogen and suboptimal carbon source) limitations, hyperosmolarity and acidic pH, stress from host innate immune attacks including reactive oxygen and nitrogen species, cationic host defense peptides (de la Fuente‐Nunez *et al*., 2013; Fang *et al*., 2016), and eventually antibiotic‐induced stresses. A variety of stress responses are known, including the broadly conserved stringent stress response, the RpoS‐mediated general stress response, the SOS DNA damage stress response, the oxidative stress response, the cell wall stress response, quorum sensing, etc. (Poole, 2012). The adaptation process frequently involves the formation of local biofilms as an alternative growth state to cope with such stresses.

Adaptation to the host environment by bacteria leads to recalcitrance to multiple therapeutic agents and thus requires novel treatment strategies in order to outsmart these complex survival strategies. Targeting effector proteins that are important in adaptation under host‐like conditions has the potential to allow us to overcome resistance towards host defenses and antibiotic treatment in such stressful environments (Dam *et al*., 2018). Thus, pathogen vulnerability to antibiotics might be induced by blocking stress‐coping defenses, using strategies that can be applied adjunctively to block disease progression. We envision that a detailed understanding of microbial stress responses and their impact on virulence and resistance to anti‐infective strategies will enable novel, more efficient ways to fight bacterial pathogens in the years to come.

We previously showed that a broad range of pathogens can be targeted and sensitized to conventional antibiotic therapy, in a cutaneous mouse high density bacterial infection (abscess) model by attacking the bacterial stringent stress response using novel synthetic peptides (Mansour *et al*., 2016; Pletzer *et al*., 2017, 2018). The highly conserved stringent stress response entails global redeployment of cellular and metabolic functions (reducing macromolecular synthesis and increasing stress coping) to promote bacterial adaptation to stress and starvation (Fang *et al*., 2016). Accumulation of guanosine tetraphosphate and/or pentaphosphate ((p)ppGpp) is largely responsible for the induction of the stringent stress response (Pletzer *et al*., 2016; Irving and Corrigan, 2018). The stringent response also regulates a form of adaptive resistance termed persistence, based on the slow growth or stasis of such organisms in the presence of antibiotics (Cabral *et al*., 2018,b). In addition, since the bacterial stringent stress response is conserved in prokaryotic organisms and not present in higher organisms, its interference could provide a route to disarm or prevent infectious diseases before they become life‐threatening. Although master regulators of virulence are promising drug targets, the effects of inhibiting them are unknown and in principle could even worsen disease prognosis. A detailed mechanistic understanding of the downstream consequences associated with blocking a master regulator during infection is warranted to fully justify the potential of this approach.

One possible direction forward would be to focus on identifying key effector proteins of bacterial stress responses, since relatively little is known about signalling that occurs downstream of key mediators, despite accumulating evidence that inhibition enhances antibiotic activity (Lee *et al*., 2009; Tkachenko, 2018). Such approaches might be limited if they are pathogen‐specific which, while novel, is counter to the general concept of relatively non‐specific antibiotics and would require ultra‐rapid diagnostics to enable their use given how rapidly most bacterial pathogens grow. Nevertheless, such compounds might be useful as adjuncts to conventional antibiotic therapy or in longer term infections where resistance arises over time.

An example of addressing stress effectors is based on the observation that mutants deficient in ppGpp production show reduced antioxidant activity in *Escherichia coli* and closely related Enterobacteriaceae (Dam *et al*., 2018). Moreover, proteins that play a key role in oxidative processes are proposed to confer stringent response‐mediated multidrug tolerance. One of these, SodB, an iron co‐factored superoxide dismutase that detoxifies intracellular superoxide radicals to oxygen and hydrogen peroxide, is a newly identified effector protein of the stringent response (Martins *et al*., 2018). Targeting SodB could lead to potentiation of a broad‐spectrum of antibiotics since its expression (likely increased in the presence of phagocytic cells *in vivo*) mediates protection against gentamicin, ofloxacin and meropenem. Additionally, SodB represents an underexplored link between membrane permeability and antibiotic internalization across several clinically relevant pathogens such as *Campylobacter jejuni*,* Staphylococcus aureus* and *Pseudomonas aeruginosa* (Hwang *et al*., 2013; Ladjouzi *et al*., 2013; Martins *et al*., 2018). Superoxide dismutase activity also correlates with expression of the phosphate‐responsive PhoB regulon, and functions to scavenge excess hydrogen peroxide in phosphate‐replete environments (Moreau *et al*., 2001). Inadequate scavenging of reactive oxygen species such as hydrogen peroxide is proposed to lead to their interaction with reactive nitrogen species, resulting in the synthesis of highly toxic metabolites such as peroxynitrate. YtfK is a nitrate‐dependent regulator of the PhoB regulon that prevents the synthesis of such toxic molecules and a newly identified effector protein that abrogates bacterial stress during *E. coli* infection (Iwadate and Kato, 2017). YtfK deletion enhances the activity of the redox‐recycling drug menandione, which triggers intracellular production of reactive oxygen species and acts synergistically with antibiotics through membrane permeabilization (Garcia *et al*., 2013). In addition to modulation of bacterial peroxidase activity, YtfK stimulates expression of metabolically important catalases such as KatG. Though more rigorous mechanistic analyses are warranted to determine the potential of these effector proteins as targets for therapeutic development and rule out host‐directed activity, their effects on susceptibility provide encouragement.

Recently, more global approaches have been identified for dismantling the bacterial virulence program, such as the disruption of microbial communication, that is, quorum sensing (QS). Bacteria use signalling molecules to inform community members of prospective environmental stressors including nutrient limitations created by increased bacterial density. The triggered signal cascade leads to rapid changes in expression of genes pertinent, for example, to virulence factor production and biofilm formation (Rutherford and Bassler, 2012; Papenfort and Bassler, 2016). Targeting QS regulatory systems, such as the autoinducer molecules acyl homoserine lactone (AHL), to out‐compete, inhibit or block signalling has been extensively studied (Givskov *et al*., 1996). These studies exemplify the feasibility of developing anti‐virulence drugs that target bacterial QS systems. Drugs that have been examined include synthetic antibodies as well as agonists and antagonists of autoinducers (Rutherford and Bassler, 2012; Reuter *et al*., 2016; Maille *et al*., 2017). Moreover, quorum quenching (QQ) enzymes that are able to degrade signalling molecules, such as AHL‐degrading lactonases and acylases, (Dong *et al*., 2001; Lin *et al*., 2003) reduce the production of harmful virulence factors thereby inducing antimicrobial vulnerability (Remy *et al*., 2018). Despite extensive testing in animal models and promising results, only a few QS inhibitors have reached human trials (Remy *et al*., 2018) with no approved drugs to date. Thus, it remains to be seen whether QQ enzymes or QS inhibitors are realistic candidate therapies for the prevention and treatment of infectious diseases.

Nevertheless, inhibition of select QS signalling pathways has interesting consequences. Terrein, a novel inhibitor of QS receptors LasR and RhlR, is associated with reduced production of virulence factors by *P. aeruginosa*. Intriguingly, terrein also influences metabolism of 3,5‐cyclic diguanylic acid (c‐di‐GMP), which leads to reduced biofilm formation (Kim *et al*., 2018). Unfortunately, terrein only showed moderate anti‐virulence efficacy in a murine airway infection model so it has limited therapeutic potential. M64, a non‐ligand‐based benzamide‐benzimidazole compound, was identified through whole‐cell high‐throughput screening and demonstrably binds to and inhibits the QS virulence regulator MvfR (PqsR) in *P. aeruginosa* (Starkey *et al*., 2014; Kitao *et al*., 2018). Intriguingly, M64 also reduces virulence and persistence in a mouse burn and lung infection model (Starkey *et al*., 2014). Additionally, M64, which has been further optimized by Spero Therapeutics, Inc. (Schutz and Empting, 2018) interferes with the activity of PqsBC, thus with the synthesis of the QS molecules 4‐hydroxy‐2‐heptylquinoline (HHQ) and 2‐heptyl‐3,4‐dihydroxyquinoline (PQS, *Pseudomonas* quinolone signal). Since both HHQ and PQS are MvfR activator molecules, their depletion may also reduce biofilm formation (Maura and Rahme, 2017; Maura *et al*., 2017). A therapy that both dismantles virulence and prevents biofilm formation is particularly desirable since approximately 80% of infections involve biofilm‐forming bacteria (Belanger *et al*., 2017; Jamal *et al*., 2018).

With improved *in silico* modelling, it is now possible to analyse high‐throughput data efficiently, which accelerates identification of candidate drug targets. Dandela *et al*. (2018) used an affinity‐based photo‐crosslinking probe to enable proteome‐wide mapping of quinolone binding proteins. Interestingly, their method identified several novel virulence factors that are part of a quinolone interactome in *P. aeruginosa* including proteins involved in lipopolysaccharide biosynthesis, as effectors for c‐di‐GMP signalling, and in the Type VI secretion machinery. In another large‐scale screen of a molecular library containing 1,600 FDA‐approved drugs, clofoctol was found to inhibit the *pqs* system and *P. aeruginosa* virulence program, most likely by targeting MvfR (D'Angelo *et al*., 2018). Although further mechanistic investigation is warranted, clofoctol, which is an approved treatment for pulmonary infections by Gram‐positive bacteria, exhibits promise as an anti‐virulence agent against *P. aeruginosa*.

Targeting effectors rather than signalling molecules of the QS system is a more specific means of fighting bacterial infections. Daly *et al*. (2015) used a sub‐inhibitory concentration of Ω‐hydroxyemodin (OHM) to block the accessory gene regulator (*agr*) signalling pathway, which controls virulence factor production in *S. aureus*. OHM bound to and inhibited the response regulator AgrA, which prevents activation of the *agr* operon. Reduced *agr* activation led to the attenuated expression of the *agr* effector RNA‐III that regulates a broad range of virulence factors in addition to phenol‐soluble modulin alpha (*psm*α) and α‐haemolysin, thereby ameliorating QS. OHM treatment also effectively reduced dermonecrosis, bacterial load, and cytokine transcription and expression in an *in vivo* model of skin infection (Daly *et al*., 2015). We have shown that we can directly sequester *psm*α, α‐haemolysin and other *S. aureus* secreted toxins by treatment with sphingomyelin or a combination of cholesterol and sphingomyelin liposomes (Wolfmeier *et al*., 2018). We applied this liposomal therapy *in vivo* in a *S. aureus* skin infection model and found that our therapeutic intervention reduced tissue dermonecrosis without affecting bacterial growth. Therefore, neutralization of secreted toxins during infection has the potential to prevent disease progression.

Prior to serious consideration of compounds as candidate therapeutics, they must show *in vivo* efficacy, slow or no resistance development, and minimal or no toxicity versus host systems. Compounds that do not meet these stringent criteria are prone to failure in the drug development pipeline. Dismantling bacterial virulence, especially under stressful conditions, is a worthwhile endeavour for future research. Future virulence‐directed therapeutic strategies may not serve as a stand‐alone antimicrobial regime but require the help of conventional antibiotics. Improving our understanding of the molecular mechanisms underlying these approaches is essential for full elucidation of their effects on the bacterial virulence program and, equally important, the human host.

## Conflict of interest

Anti‐biofilm peptides including ones discussed here were invented in part by REWH, assigned to his employer the University of British Columbia, filed for patent protection and licenced to ABT Innovations Inc. in which Hancock has an ownership position
